# Prevalence and associated factors of common mental disorders among residents of Illu Ababore zone, southwest Ethiopia: a cross-sectional study

**DOI:** 10.1186/s13033-020-00394-3

**Published:** 2020-08-12

**Authors:** Nigus Alemnew Engidaw, Zakir Abdu, Ishwari Chinani

**Affiliations:** 1 College of Health Sciences, Debre Berhan University, P.O. Box 445, Debre Berhan, Ethiopia; 2 Faculty of Public Health and Medical Science, Mettu University, Mettu, Ethiopia

**Keywords:** Common mental disorder, Prevalence, Ethiopia

## Abstract

**Background:**

A common mental disorder is characterized by anxiety, depression, and unexplained somatic symptoms that usually encountered in community and primary care settings. Both short and long term bio psychosocial disabilities are inevitable if common mental disorder is not treated. Despite its impact, the prevalence of common mental disorder in the Illu Ababore zone is not well known. Therefore, this study aimed to assess the prevalence and associated factors of common mental disorder among Ilu Ababore zone residents, Southwest Ethiopia.

**Method:**

A community based cross-sectional study was conducted from July 1 to August 30, 2018. A multi-stage sampling technique was applied to recruit participants. Self-Reporting Questionnaire (SRQ-20) was used to assess the presence of common mental disorder. The data were entered into Epidata version 3.1 and analyzed by using SPSS version 23 software. Bivariate and multivariate binary logistic regressions were computed to identify the associated factors. Statistical significance was considered at *P* value < 0.05.

**Result:**

A total of 690 participants were enrolled in this study with a response rate of 91.39%. The prevalence of common mental disorder was 27.2% (95% CI, 23.9, 31.0%). Being female (AOR = 1.76, 95% CI = 1.15, 2.69), unable to read and write (AOR = 3.06, 95% CI = 1.37, 6.82), living in the rural area (AOR = 3.53, 95% CI = 2.01, 6.18), having a family member with mental illness (AOR = 2.68, 95% CI = 1.6, 4.5), having a chronic physical illness (AOR = 3.48, 95% CI = 2.26, 5.34) and lifetime alcohol use (AOR = 4.55, 95% CI = 2.93, 7.0) had a significant association with common mental disorder.

**Conclusion:**

The current study showed that the proportion of the common mental disorder was high. Females showed a higher prevalence of the common mental disorder. Having a chronic physical illness, resides in the rural areas and history of lifetime alcohol use were also significantly associated with CMD. Psychological and social interventions with greater emphasis on females who have low educational status and residing in the rural area are recommended. Strategies that focus on the proper treatment of chronic physical illness can be also helpful to minimize the occurrence of common mental disorder.

## Introduction

Mental disorder is the most common challenge for both current and future generations will face [1]. One in four people develops a mental disorder during their lifetime [2]. One of the most prevalent mental disorders in the world is a common mental disorder (CMD) [3]. CMD is a group of mental disorder which manifest with anxiety, depression, and unexplained somatic symptoms [4–6]. It usually encountered in community and primary care settings [5].

Recently, CMD is responsible for 14% of the total burden of disease. By the year 2030, it is predicted to be the first leading cause of disease burden [7]. It is reported to occur with more frequency among women than men, especially in low and middle- income countries [8, 9].

A study conducted in England revealed that one in six adults had a CMD [8]. A systematic review and meta-analysis study at the University of New South Wales indicated 29.2% of CMD across their lifetime [10]. The estimated prevalence of CMD in Brazil was 29.3% [11]. The studies in Tanzania showed that the prevalence of CMD was 24–28.8% [9, 12]. In different regions of Ethiopia, the prevalence of CMD ranges from 14.9 to 33.6% [13–15].

If CMD is left untreated, both short and long term physical, social, and occupational disabilities are inevitable [8]. The long term effects of CMD is due to the relapsing nature of the problem, poor adherence, and treatment-seeking behavior [7]. CMD is associated with disability, poor prognosis of comorbid diseases, impacts on health care costs, and economic productivity [16]. From the community, the most affected groups are young ages, females, and singles. Lower socioeconomic status, psychological illnesses, poor reproductive health, gender disadvantage, and physical ill-health are the main risk factors for this disorder [5].

The effects of CMD on the general population are relatively well studied in developed countries, but there have been fewer studies in low and middle-income countries especially in Ethiopia. Little has been investigated about the prevalence of CMD and associated factors among the general community at the national level. In our study area, there is no study conducted on CMD. Therefore, the current study aimed to assess the prevalence of common mental disorder and its associated factors among residents of the Ilu Ababore zone. It will be vital for the zone health department to address community mental health issues. Besides, it will be an input for policymakers and planners to indicate appropriate measures to tackle the problem regarding common mental disorders in the community.

## Methods

### Study area

The study was conducted in the Ilu Ababore zone which is one of the administration zones in Oromia regional state. The zone is located in the southwestern part of Ethiopia which is 600 kms away from the capital city of Ethiopia, Addis Ababa. Illu Ababore zone has 12 administrative woredas. Based on the 2007 Census conducted, the Illu Ababore zone has a total of 1,271,609 men and 634,623 women population. The zone has an area of 15,135.33 square kilometers, while 124,428 or 12.16% are urban inhabitants. A total of 272,555 households were counted in this Zone, which results in an average of 4.67 persons to a household, and 263,731 housing units. The zone has two hospitals and 20 health centers. Psychiatry service has been giving at the outpatient department of Mettu Karl referral hospital. None of the hospitals in the Illu Ababore zone are providing inpatient psychiatry service (unpublished Illu Ababore zone health office report 2018).

### Study design and period

A community-based cross-sectional study design was carried out from July 1 to August 30, 2018.

#### Source and study population

All permanent residents of the Illu Ababore zone aged ≥ 18 years were the source population. The study population was a sample of adult residents living in Illu Ababore zone. Individual residents who were seriously ill and those who had hearing and speech impairment were excluded from this study.

### Study variables

The dependent variable was a common mental disorder (CMD). Independent variables included sociodemographic factors (age, sex, marital status, ethnicity, residence, household type, religion, educational status and occupational); clinical factors (having chronic physical illness); substance-related factors (lifetime alcohol, khat, and cigarette use) and psychosocial factors (family member with mental illness and social support).

### Sample size determination

The sample size was determined using a single population proportion formula. It was calculated with the assumptions of 33.6% prevalence of common mental disorder from the study done in Jimma town [15]. 0.336 P, 1.96 Z (standard normal distribution), 95% CI, ⍺ = 0.05. Considering the design effect and adding a 10% non-response rate, the final sample size was 755.

#### Sampling technique and procedure

A Multistage sampling technique was used to select a representative sample. A sampling frame of all the woredas in the Illu Ababore zone was drawn and stratified into urban and rural areas. From a total of 12 woredas in the Illu Ababore zone, only four woredas were selected due to logistic constraints. Accordingly, Hurumu, Algesachi, Yayo, and Bure woredas were selected by the lottery method. Six urban and nineteen rural kebeles were obtained by simple random technique. The samples were allocated to each kebeles proportionally based on the household size of the kebeles. A systematic sampling technique was applied to select households. The first household was selected randomly between 1st and kth. Study subjects in every kth household were interviewed. When there were more than one study subject in one household, a lottery method was used to select a participant. Accordingly, a total of 755 participants were invited to participate in the study.

### Data collection instruments and procedures

The data were collected by face-to-face interviews using semi-structured questionnaires which consist of socio-demographic factors, clinical characteristics, Oslo 3 item social support scale, substance-related factors, and self-reported questionnaire (SRQ-20). The outcome variable (common mental disorder) was assessed by using SRQ-20. The SRQ was originally designed by WHO as a self-administered scale. Because of the low literacy rate in developing countries, SRQ-20 was also found to be suitable for an interviewer-administered questionnaire [17]. Each of the 20 items is scored 0 or 1. A score of 1 indicates that the symptom was present during the past month, a score of 0 indicates that the symptom was absent. Participants who scored 8 and more in SRQ-20 in the past 4 weeks were considered as having CMD [17]. SRQ-20 was validated in low and middle-income countries [18, 19]. Social support was measured by Oslo 3-item social support scale. Individuals who scored 3–8, 9–11, and 12–14 were considered as having poor, moderate, and strong social support, respectively [20, 21]. The magnitude of substance like lifetime alcohol use, nicotine use, and khat chewing was assessed by a developed structured questionnaire.

The data were collected by face to face interviews using a pre-tested questioner. We used thirteen health extension workers (Diploma nurses) and two mental health professionals (MSc in integrated clinical and community mental health) as data collectors and supervisors respectively. A 2 days training was given for the data collectors on the purpose of the research, the contents of the data collection instruments, ethical issues, and how to approach study participants. The supervisors were trained to oversee participant recruitment, data collection, checking, and controlling data quality. The data- collection process was closely followed-up by the supervisors.

### Data quality control issues

The questionnaire was translated into Afaan Oromo (local language) by Afaan Oromo speaking linguist and back translation to English was performed by mental health specialists. The Afaan Oromo version of the questionnaire was pre-tested on 5% of the total study participants at Mettu town to check the consistency and length of time each questioner took. The prepared questionnaire was checked thoroughly for its completeness, objective, and variable before it was interviewed by data collectors.

### Data processing and analysis

All the collected data were checked for completeness and consistency and entered into Epidata version 3.1 statistical software and then exported to SPSS windows version 23 program for analysis. Descriptive statistics were computed to explain the socio-demographic characteristics, clinical variables, psychosocial characteristics, and CMD. Bivariate and multivariate logistic regression analyses were done to identify the relationship between the dependent and independent variables. Variables that have *p *value < 0.20 in the bivariate model were entered into the multivariate analysis to avoid potential confounders. In the multivariate model, variables with *P* values of less than 0.05 were considered as statistically significant predictors of depression. The results were presented in text and tables with adjusted odds ratio (AOR) and the corresponding 95% confidence interval.

## Result

### Socio-demographic characteristics of respondents

From a total of 755 individuals invited to participate in this study, 690 participants completed the interview properly with the response rate of 91.39%. Among the respondents, 420 (60.8%) were males. The majority (27%) of the respondents were in the age range of 18–25 years. Two hundred ninety-eight (43.1%) of the participants were Orthodox followers and more than 2/3rds (65.7%) were single. Concerning the educational status of the respondents, 220 (31.9%) of the participants attended primary school education (grade 1–8). About 3/4th (73.9%) were living in a rural area and 584(84.6%) of the participants live with their family (Table 1).

**Table 1.** Sociodemographic characteristics of study participants among adults of Ilu Ababore zone, Southwest Ethiopia, 2018 (n = 690)

Study variables	Frequency (N)	Percentage (%)
Gender		
Male	420	60.8
Female	270	39.2
Age		
18–25	186	27.0
26–25	156	22.6
31–39	168	24.3
≥ 40	180	26.1
Marital status		
Married	194	28.1
Single	454	65.8
Divorced	24	3.5
Widowed	18	2.6
Religion		
Muslim	114	16.5
Orthodox	298	43.2
Protestant	278	40.3
Residence		
Rural	510	73.9
Urban	180	26.1
Educational status		
Able to read and write	108	15.7
Can read and write	82	11.9
1–8 grade	220	31.9
9–10 grade	196	28.4
10 + and above	84	12.2
Having job		
Yes	632	91.6
No	58	8.4
Living arrangements		
With family	584	84.6
Alone	106	15.4

### Clinical factors, social support, and Lifetime substance use characteristics of study participants

In this study, 214 (31.01%) of respondents had a chronic physical illness. One in every six (15.9%) of the respondents had a family history of mental illness. Thirty-two (4.6%) of the respondents had a history of past psychiatric illness. Nearly 1/5th (22.7%) of the respondents took alcohol at least once in their lifetime. Nearly half (49.4%) of respondents reported that they have moderate social support and 24 (3.5%) of respondents had history lifetime cigarette smoking. The study showed that nearly 1/5th (22.0%) of respondents’ chewed khat at least once in their lifetime (Table 2).

**Table 2:** Clinical factors, social support and lifetime substance use characteristics of study participants among adults of Ilu Ababore zone, Southwest Ethiopia, 2018 (n = 690)

Study variables	Frequency (N)	Percentage (%)
Family member with mental illness		
Yes	110	15.9
No	580	84.1
Chronic physical illness*		
Yes	214	31.01
No	476	68.98
Past psychiatric illness		
Yes	32	4.6
No	658	95.4
Social support		
Poor support	106	15.4
Moderate support	341	49.4
Strong support	243	35.2
Alcohol use (lifetime)		
Yes	157	22.7
No	534	77.3
Khat use (lifetime)		
Yes	152	22.0
No	538	78.0
Cigarette smoking (lifetime)		
Yes	24	3.5
No	666	96.5

*Heart disease, Hypertension, Diabetes mellitus, Epilepsy, HIVV/AIDS, Asthma

### Prevalence of common mental disorder and associated factors

In this study, the prevalence of CMD was found to be (27.2%) with 95% CI: (23.9–31.0%). It was higher in females (35.1%) than in males (19.3%). In bivariate analysis; being female, unable to read and write, living in a rural area, having a family member with mental illness, having a chronic physical illness, being age above 40 years, previous history of mental illness, lifetime use of alcohol, khat chewing, and cigarette smoking were associated with CMD.

In the multivariate model, variables with P-values of less than 0.05 were considered as statistically significant predictors of CMD. Accordingly, being female, unable to read and write, living in a rural area, having a family history of mental illness, having a chronic physical illness and lifetime alcohol use had a significant association with CMD. The odds of having common mental disorder among female participant was 1.76 times higher (AOR = 1.76, 95% CI = 1.15, 2.69) than male. Those who could not read and write were 3.06 times more likely to develop CMD (AOR = 3.06, 95% CI = 1.37, 6.82) compared with those who were educated grade 10 and above. The odds of having CMD among participants who lived in a rural area was 3.53 times (AOR = 3.53, 95% CI = 2.01, 6.18) higher than those who reside in the urban area. Participants who have family members with mental illness were 2.68 times more likely to develop CMD (AOR = 2.68, 95% CI = 1.6, 4.5) as compared with their counterparts. Those who had a chronic physical illness were 3.48 times (AOR = 3.48, 95% CI = 2.26, 5.34) were more likely to have CMD than those who had no chronic physical illness. The odds of having CMD among participants who were lifetime alcohol use was 4.56 times (AOR = 4.55, 95% CI = 2.93, 7.0) than participants who did not use alcohol in their lifetime. (Table 3).

**Table 3:** Bivariate and multivariate logistic regression analysis of common mental disorders and associated factors among adults of Ilu Ababore zone, Southwest Ethiopia, 2018 (n = 690)

Study variables	CMD		COR(95% CI)	AOR(95% CI)
	Yes N (%)	No N (%)		
Genderb^b^				
Male	130 (31.0)	290 (69.0)	1	1
Female	58 (21.5)	212 (78.5)	1.64 (1.15, 2.34)**	*1.76 (1.15, 2.69)*
Age (year)				
18–25	42 (22.6)	144 (77.4)		1
26–30	40 (25.6)	116 (74.4)	1.18 (0.72, 1.94)	1.37 (0.76, 2.47)
31–39	44 (26.2)	124 (73.8)	1.21 (0.75, 1.98)	1.11 (0.62, 2.0)
40 and above	62 (34.4)	118 (65.6)	1.8 (1.13, 2.86)*	0.80 (0.44, 1.46)
Residence^b^				
Rural	162 (31.8)	348 (68.2)	2.75 (1.45, 4.35)**	*3.53 (2.01, 6.18)*
Urban	26 (14.4)	154 (85.6)	1	1
Educational status				
Unable to read and write^b^	48 (44.4)	60 (55.6)	3.40 (1.75, 6.60)**	*3.06 (1.37, 6.82)*
Can read and write	14 (17.1)	68 (82.9)	0.87 (0.39, 1.9)	0.62 (0.24, 1.61)
1–8 grade	52 (23.6)	168 (76.4)	1.31 (0.70, 2.46)	1.07 (0.51, 2.26)
9–10 grade	58 (29.6)	138 (70.4)	1.79 (0.95, 3.33)	1.78 (0.84, 3.76)
10 + and above	16 (19.0)	68 (81.0)	1	1
Family member with mental illness				
Yes	62 (56.4)	48 (43.6)	4.65 (3.04, 7.12)**	*2.68 (1.60, 4.50)*
No	126 (21.7)	454 (78.3)	1	1
Chronic physical illness^b^				
Yes	98 (45.8)	116 (54.2)	3.62 (2.54, 5.16)**	*3.48 (2.26, 5.34)*
No	9 0(18.9)	386 (81.1)	1	1
Past mental illness				
Yes	18 (56.3)	14 (43.7)	3.69 (1.8, 7.58)**	0.99 (0.41, 2.42)
No	170 (25.8)	488 (74.2)	1	1
Alcohol use (life time)^b^				
Yes	8 3(52.9)	74 (47.1)	4.57 (3.13, 6.68)**	*4.55 (2.93, 7.0)*
No	105 (19.7)	428 (80.3)	1	1

Italic values indicate significance of *p* value (*p* < 0.05)

*p < 0.05, **p < 0.001(in multivariate model)

## Discussion

The objective of this study was to assess the prevalence and associated factors of common mental disorder among Ilu Aba bora zone residents, southwest Ethiopia.

The current study showed that the prevalence of CMD among residents of the Illu Ababore zone was 27.2%. The finding was in line with studies carried out in Brazil (29.3–30.0%) [4, 11] and Tanzania (28.8%) [12]. However, it was higher than the study done in England (17.0%) [8], University of New South Wale (17.6%) [10], Tanzania (15%) [22], Kenya (12%) [23] and Ethiopia (14.9–17.7%) [13, 24]. The probable reason for the difference might be due to the differences in screening tools, study design, study period, the difference in socio-economic characteristics, and health service supply. For example, the study in England has used the revised clinical interview schedule(CIS-R) to assess the presence of common mental disorder [8] and in the study of New South Wale, a meta-analysis was used [10]. The other probable reason for the difference could be; in developed countries like England, they may supply better mental health services for their community. The participants in developed countries may have better access to education, mental health information, and utilization [25, 26]. Studies have suggested that poor education is a consistent risk factor for common mental disorders and poor mental health can put educational attainment at risk [27, 28]. The mental health service in our study area is not sufficient. In the Illu Ababore zone, only one hospital has been giving outpatient psychiatry services. Inpatient mental health services are not available at any of the health facilities in the zone. This shows the need for empowering the community by providing adequate mental health information and by expanding mental health services in Illu Ababore zone.

On the contrary, our finding was lower than the study done in Brazil (33.7%–44.9) [29, 30], South Africa (64.5%) [22], and Ethiopia 63.1% [3]. The variation might be due to the difference in screening tools and cutoff points, the difference in the study population, and study setting. In the study of South Africa, they used CIS-R to screen common mental disorders [22]. The Kessler psychological distress (K10) scale was used in a study done in Ethiopia among university students [3]. While the current study conducted among the general population, the above study participants' were only students and young and middle age groups [29, 30]. Our study was carried out both in the urban and rural setting, while the above studies were conducted only in urban setting.

Regarding the associated factors of CMD, female participants were 1.76 more likely to developed CMD compared to males. Similar findings were reported in the previous studies done in Brazil [4], Tanzania [22], and Kenya [23]. This might be due to the pressures created by their multiple roles and responsibilities, gender discrimination, and gender-based violence. In most Ethiopian society especially in rural areas, women are expected to stay at home to care for their children and other home activities. In addition, women had a limitation of activities outside their homes, fewer opportunities for them in education and employment, and greater risk of domestic violence [14]. The other reason might be; women faced different hormonal changes at some stages of their life such as during menstruation cycle, pregnancy, and menopause which may predispose them to emotional changes.

Participants, who could not read and write were more likely to develop CMD than those who attended grades 10 and above. It is supported by studies conducted in northeast Ethiopia and Tanzania [9, 28, 31]. Having low education status may lead to a reduced chance to access resources to improve their living condition including their mental health situation. As the literature suggested, low educational status has a relationship with mental disorders [32]. Low educational status prevents access to most professional jobs, increases vulnerability and insecurity, and contributes to a persistently low social capital [28]. In our study, only 12.2% of the participants attended grade 10 and above. This indicates the advantage of expanding education to minimize the occurrence of CMD.

Participants who had a family member with mental illness were 2.68 times more likely to develop CMD as compared with their counterparts. Studies have been suggested that those with a family history of mental illness are more prone to develop CMD. Having a family history of a mental disorder increases the risk of developing a mental illness in the long run of a person's life [8, 33]. This may be due to the genetic base of mental illness.

The odds of developing CMD among those who had a chronic physical illness were 3.48 times more than those who had no known chronic physical illness. It is consistent with studies that were conducted previously [4, 5, 8, 22, 23, 29, 34]. This might be due to the following reasons. One, the direct effect of the illness and the side effects of the drugs which are being prescribed for the illness. Second, the effect of illness comorbidity may affect the treatment outcome, help-seeking behavior, and compliance to treatment. Besides, a decreased ability to function is common in individuals with chronic physical illness [5].

Those who had a history of lifetime alcohol use were 4.5 times higher to develop CMD. It is supported by previous studies [3, 5, 8, 9]. But inconsistent with the study conducted in Harar, eastern Ethiopia [13]. This might be explained by the fact that the study in Harar was carried in the area where alcohol was not well drunk due to religious reasons. As different studies reported, there is coexistence between CMD and different psychoactive substances including Alcohol [22]. Alcohol drinking significantly increased the risk of CMD and having CMD by itself may lead to drinking alcohol to alleviate their symptoms [3].

The odds of developing CMD is more than three times for participants who reside in the rural area. The current study was inconsistent with previous studies carried out in western countries [35]. The possible reason could be; in developing countries such as Ethiopia, there is a lack of mental health services, especially in rural areas. In our study area, most of the community resides in the rural area and mental illness service is rarely utilized by the community due to different reasons. First, due to a lack of awareness about mental illness. Second, the preference for traditional treatment for the treatment of mental illness in most of the society in Ethiopia. This indicates the need for expanding mental health services to the rural areas of Ethiopia. In 2012, the federal ministry of health of Ethiopia has been introduced ‘‘Ethiopia’s National Mental Health Strategy’’ to address the mental health needs of all Ethiopians through quality, culturally competent, evidence-based, equitable, and cost-effective care [36]. Even though there are improvements from the previous years, lots of work is needed to expand mental health services in the rural area of Ethiopia.

The finding from this study reveals several practical applications. First, it would help the zone health sector to design more effective programs in the management of common mental disorders. Second, our study result will be used as a baseline for researchers who want to conduct national-level studies regarding the burden and prevention of common mental disorders among adults in Ethiopia.

### The strength and limitation of the study

The use of relatively adequate sample size and standardized and reliable tools were the strength of this study. However, the current study also had some limitations: first, due to the cross-sectional nature of the study the association between different factors and common mental disorders may not imply causation; second, the study has no access to detailed previous psychiatric records or collateral information. Third, chronic physical illness was assessed by self-reported responses.

## Conclusion

In conclusion, CMD among residents of Ilu Ababore zone was high. Females showed higher CMD than males. Those who could not read and write and residing in the rural area and were significantly associated with CMD. Having chronic physical illness and history of lifetime alcohol use were also significant predictors of CMD. Psychological and social interventions with greater emphasis on females who have low educational status and having chronic physical illness is recommended. Strategies that focuses on the treatment of chronic physical illness and cutting alcohol drinking can be helpful to minimize the occurrence of CMD.

## Data Availability

The datasets generated and/or analyzed during the current study are not publicly available due to some privacy reasons, but part of the row datasets will be available in the recommended publicly available data repository of BMC or from the corresponding author on reasonable request.

## Abbreviations

AOR: Adjusted odds ratio
CI: Confidence interval
CIS-R: Clinical interview schedule
CMD: Common mental disorder
COR: Crude odd ratios
WHO: World Health Organization
